# Hematopoietic Stem Cells (HSC) and Granulocyte Macrophage Progenitors (GMP) are the Oxidative Stress Targets in Low/Intermediate-1 Risk Myelodysplastic Syndromes

**DOI:** 10.26502/jbb.2642-91280063

**Published:** 2022-11-09

**Authors:** Valentina Giai, Thea Bensi, Claudia Bertassello, Michela Savio, Dario Ferrero, Maria Matilde Ciriello, Marco Ladetto

**Affiliations:** 1 Division of Hematology, SS Antonio e Biagio and Cesare Arrigo Hospital, Alessandria, Italy; 2 Flow-cytometry worked, SS Antonio e Biagio and Cesare Arrigo Hospital, Alessandria, Italy; 3 Pathology, SS Antonio e Biagio and Cesare Arrigo Hospital, Alessandria, Italy; 4 Division of Hematology, University of Turin, Torino, Italy; 5 Division of Hematology, AUO Città della Salute e della Scienza, Torino, Italy

## Abstract

**Introduction:**

Myelodysplastic Syndromes (MDS) are a heterogenous group of clonal hematopoietic stem cell malignancies. Previous studies showed that Reactive Oxygen Species (ROS) play a role in the pathogenesis and clinical evolution of MDS, contributing to Hematopoietic Stem and Progenitor Cells (HSPC) genetic instability. Less is known about ROS levels in the various sub-populations of MDS HSPC and how they correlate with clinical data in MDS patients. Our study aims to analyze ROS levels in MDS Hematopoietic Stem Cells (HSC), common myeloid progenitors (CMP), Granulocyte Macrophages Progenitors (GMP) and megakaryocyte-erythrocyte progenitors (MEP); afterwards, we looked at the relationship between ROS levels and clinical data.

**Methods:**

thirty-eight MDS and 27 Normal Bone Marrow (NBM) samples were collected; ROS levels were analyzed via multicolor flow cytometry.

**Results:**

In both NBM and MDS, HSC showed much higher ROS levels than progenitors (3 to 4 folds, p < 0.0001); HSC ROS were significantly more elevated in MDS-no excess blasts versus MDS with excess blasts and versus NBM. GMP from MDS-no excess blasts showed higher ROS compared to NBM GMP. The 3 MDS with Ringed Sideroblasts (RS) showed more elevated ROS in HSC and GMP compared to the not RS low/intermediate-1 MDS; the 2 monosomy 7 patients displayed higher ROS levels in each subpopulation compared to the normal karyotype MDS; the only del(5q) patient did not show relevant differences in ROS levels compared to the median of the normal karyotype MDS ROS. The 9 high transfusion burden patients exhibited higher ROS in HSC and GMP compared to NBM HSC and GMP. These data were not confirmed in low transfusion burden (n:2) and non-transfused patients (n:26). In low/intermediate-1 MDS, a direct correlation between ferritin values and ROS levels in progenitors, but not in HSC, was detected. Interestingly, low/intermediate-1 risk patients that are no longer responding to recombinant human erythropoietin (rh-EPO) showed higher ROS levels in GMP and HSC.

**Conclusions:**

Our data showed that ROS can play a role in the pathogenesis and maintenance of low and intermediate-1 risk MDS clone; ROS status can be influenced by several clinical factors as ferritin levels and rh-EPO treatment. In this scenario, high ROS levels can contribute to genetic instability and influence progression to AML. Further biological studies are needed to elucidate ROS role in MDS pathogenesis and analyze the possible benefit of antioxidant drugs added to the standard MDS treatments.

## 1. Introduction

Myelodysplastic Syndromes (MDS) are a heterogeneous group of myeloid malignancies, characterized by ineffective hematopoiesis and frequent progression to Acute Myeloid Leukemia (AML). MDS patients show peripheral cytopenia and poor clinical outcomes: median overall survival is less than 2 years in the high-risk subtype [1]. The ultimate therapeutic goal in MDS is to delay as much as possible the evolution in AML and, so, improving survival. MDS is a hematopoietic stem cell disease: rare MDS Stem Cells (MDS-SC) and their related myeloid progenitors had been described [2]. Through a flowcytometry based technique, normal and MDS Lineage Negative (Lin-) CD34+ cells can be divided in Hematopoietic Stem Cells (HSC), Common Myeloid Progenitors (CMP), Granulocytic Macrophages Progenitors (GMP) and Megakaryocyte-Erythrocyte Progenitors (MEP) [3–5]. In low and intermediate risk MDS, CMP is the most represented subpopulation; in high risk MDS, instead, GMP ratio is increased, as well as HSC, compared to lower risk MDS [6]. Within the last 10 years, next generation sequencing techniques have allowed a better insight into the genetic landscape of MDS. The most frequent mutations concern genes involved in the RNA splicing machinery, DNA methylation, chromatin modification, signal transduction, cohesin regulation and DNA repair [7, 8]. The number and type of mutations influence the prognosis of MDS patients and the probability of AML transformation [9]. Since the 2000s, the role of Reactive Oxygen Species (ROS) is under investigation in MDS. An increase of proinflammatory cytokines has been described in MDS bone marrow microenvironment, leading to an ultimate ROS increase in MDS cells. Oxidative stress affects self-renewal, proliferation and differentiation of hematopoietic cells [10]. Terminal differentiation of erythroid cells can be influenced by ROS: a recent study showed that, when enzyme Isocitrate Dehydrogenase 1 (IDH1) is downregulated or knocked out, as in some IDH1 mutated AML and MDS, the oxidative stress induces the synthesis of dysplastic erythroblasts with morphological abnormalities, as double nuclei. The addition of ascorbic acid, the so known vitamin C, can scavenge ROS and reprogramming mitochondrial normal function [11]. ROS levels are increased in red blood cells, platelets and polymorphonuclear leukocytes in MDS patients [12] as well as in MDS bone marrow mononuclear cells [13]. Peroxiredoxin-2 (PRDX2), a member of the peroxiredoxin family that regulates ROS, was found increased in granulocytes of low risk MDS patients [14]; ROS sources, such as NAD(P)H oxidase (Nox) complexes, regulate gene expression and splicing factors activity in MDS bone marrow [15]. One of the main factors contributing to oxidative stress in MDS patients is Iron Overload (IO) [16]. IO occurs mostly in low and intermediate-1 risk patients, because of the chronic transfusion burden. In these patients, oxidative stress can lead to genomic instability [17] and AML clonal evolution [18, 19]. It is well known that overall survival in MDS patients on iron chelation therapy is higher than in non-chelated MDS patients, suggesting that ROS status can influence survival [20, 21]. It is important to underline that many recurrent mutations in MDS are involved in the regulation of oxidative status in MDS microenvironment: for example, TET2 recruited histone deacetylase enzyme 2 (HDAc2) and repressed transcription of interleukin-6 (IL-6), one of the most important inflammation cytokines that promotes inflammation and ROS production [22, 23]; ASXL1 mutation causes ROS increase via NAD(P)H oxidase and consequent cell death by pyroptosis [24]. Also mutations involving spliceosomes components, such as SF3B1, SRSF2 and U2AF1, can induce hematopoietic cells death activating NF-kB pathway and IL-6 excessive release [25, 26]. The present study aims to investigate ROS levels among the different subpopulations of MDS Hematopoietic Stem (HSC) and Progenitor Cells (CMP, GMP and MEP) and correlate the results with clinical data.

## 2. Methods

Thirty-eight MDS and 27 control bone marrow samples were collected in our Institution (SS Antonio e Biagio e Cesare Arrigo Hospital, Alessandria, Italy) from September 2016 to January 2019 (Table 1).

**Figure 1:**
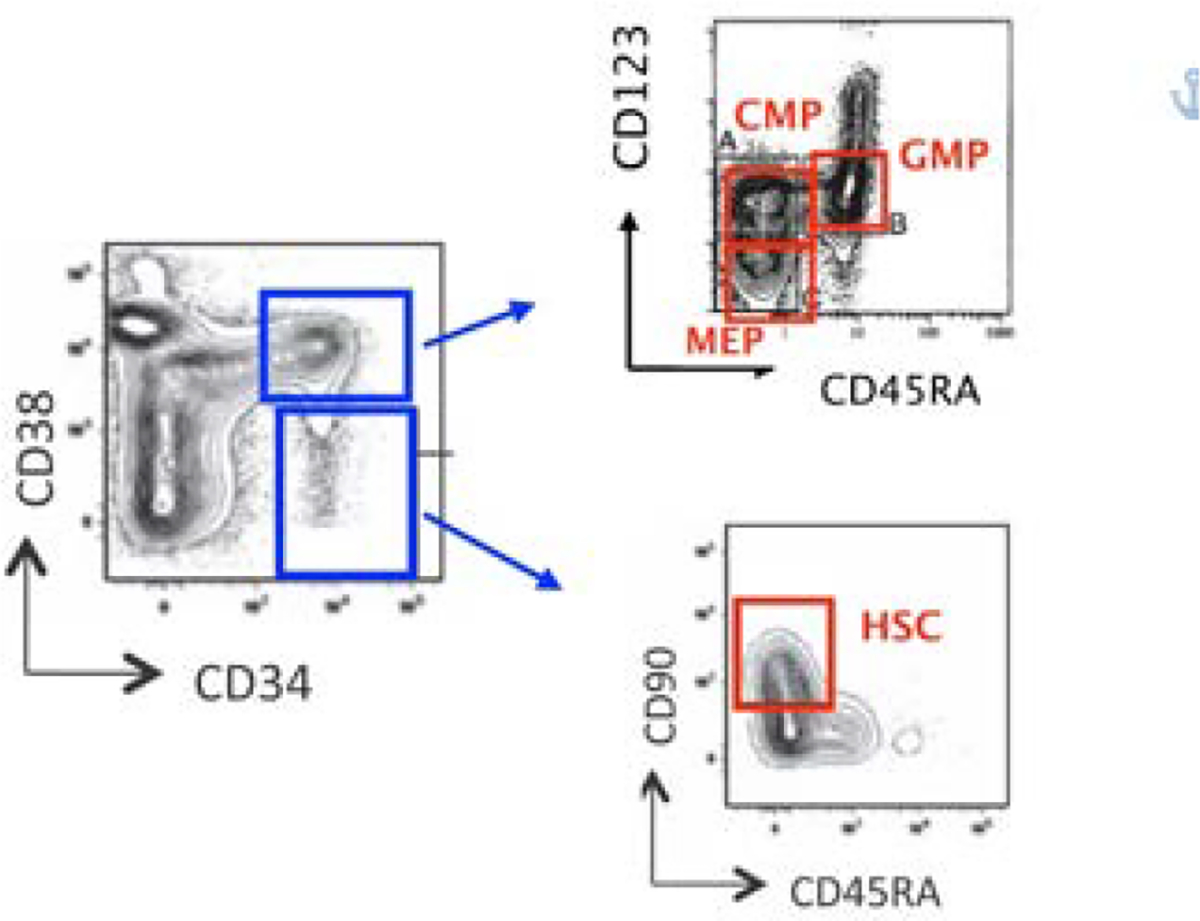
Flow cytometry gating strategies on whole bone marrow samples, without further CD34+ cells enrichment procedures. Abbreviations: CMP- Common Myeloid Progenitors; GMP- Granulocyte Macrophages Progenitors; MEP- Megakaryocyte Erythroid Progenitors; HSC- Hematopoietic Stem Cells.

MDS were categorized according to WHO 2016 classification. In the low/intermediate-1 risk MDS, 7 were on human recombinant erythropoietin (rh-EPO) therapy, 4 had discontinued this treatment by a median time of 6 months and 19 were in clinical follow up without any therapy. All samples were fetched in ethylendiamineteraacetic acid (EDTA) tubes after obtaining written informed consent. The use of human materials was approved by the Local Ethical Committee in accordance with the Helsinki Declaration. As normal controls (NBM), bone marrow aspirates from 17 patients with Non-Hodgkin Lymphoma (without lymphoma bone marrow involvement), 7 with Idiopathic Thrombocytopenia and 8 with no pathological abnormalities were used. Samples were run for analysis one hour after bone marrow aspiration, and, to avoid extra stress to cells, with no further CD34+ cells enrichment procedures. ROS levels were assessed using 2’,7’-dichlorofluorescin diacetate (DCFH2-DA; Sigma-Aldrich, Saint Louis, MO, USA). With the view to identify hematopoietic stem and progenitor cells, whole blood cells were stained and analyzed by flow cytometry, as described above [27, 3, 4, 28]. Briefly, bone marrow cells were stained with a monoclonal Antibodies (mAbs) mix for 30 minutes at 4°C in the dark. The mAbs cocktail was composed as follows: CD90-PE/CD38-PC7/CD123-APC/CD34-APC-Alexa Fluor 700/CD45RA- APC-Alexa Fluor 750, 7AAD (Beckman Coulter Life Sciences, Brea, CA, USA) and Anti Hu Lineage Cocktail-PB (CD3, CD14, CD16, CD19, CD20, CD56 – Biolegend, San Diego, CA, USA). Then, cells were incubated for 15 minutes in lysing buffer (150 mM Ammonium Cloride, 10 mM Potassium Hydrogen Carbonate, 0,1 mM EDTA, pH 7,2–7,4; Sigma-Aldrich, Saint Louis, MO, USA), and therefore they were washed twice with phosphate buffered saline (PBS, Life Technologies, Carlsbad, CA, USA) by centrifugation at 350g for 5 minutes and immediately acquired by cytometer for ROS measurements. For each sample, at least 7 million events were collected by Navios Flow Cytometer (Beckman Coulter Life Sciences, Brea, CA, USA). The results were analysed through Kaluza Analysis software (Beckman Coulter Life Sciences, Brea, CA, USA). ROS were measured on HSC and CD34+ myeloid precursors CMP, GMP and MEP. ROS levels in each cell subpopulation were expressed as mean fluorescence intensity (MFI): in order to normalize results, for each sample, we used ratio of each subpopulation MFI and related lymphocytes MFI. The gating strategy was performed as illustrated in figure 1.

For flow cytometer verification performances, as optical alignment, fluidics and laser setting, we used, at each experiment, standardized fluorospheres (Flow-Check Pro Fluorospheres and Flow-Set Pro Fluorospheres, Beckman Coulter Life Sciences, Brea, CA, USA), before sample acquisitions.

## 3. Statistical Analysis

Graphpad Prism software (San Diego, CA, USA) was used for all statistical analyses. Comparison among groups were assessed by paired *t*-tests and Anova tests. Values of P < 0.05 were considered statistically significant.

## 4. Results

**Figure 2:**
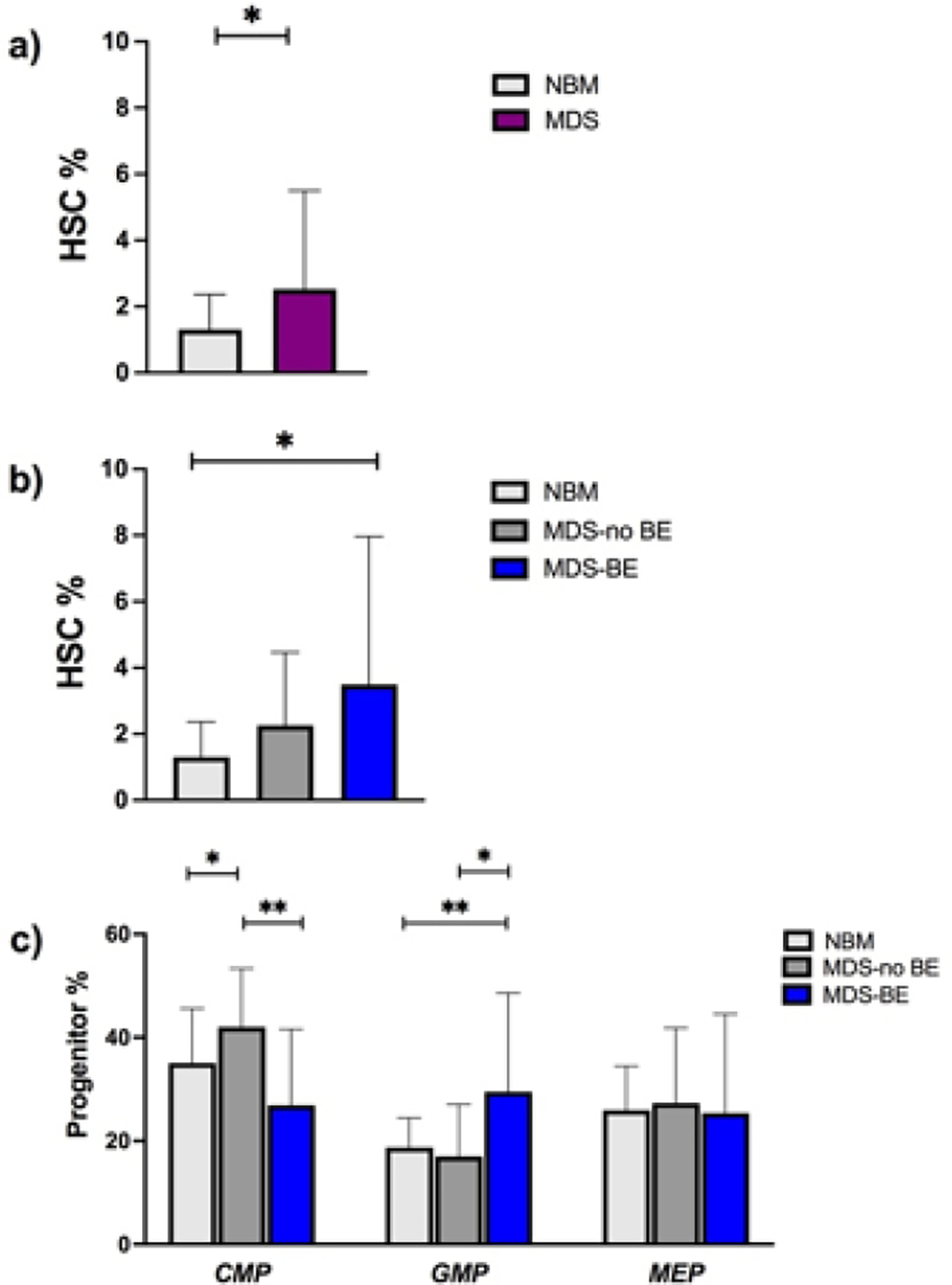
percentages of myeloid progenitors and hematopoietic stem cells in normal controls and in MDS.HSC percentages on Lin- CD34+CD38- cells were more elevated in MDS than in NBM (2.5% vs 1.3%, *p*: 0.038) (Figure 2a); in particular, MDS-EB showed a higher HSC ratio compared to NBM (3.5% vs 1.3%, *p*: 0.023) and to MDS-no EB (2,2%) (Figure 2b). Among Lineage negative (Lin-) CD34+CD38+ cells, the percentage of CMP was 42%, 26.8% and 35.7% for MDS-no EB, MDS-EB and NBM respectively (MDS-no EB vs MDS-EB *p*: 0.003). GMP were 17%, 29.5% and 18.7% for MDS-no EB, MDS-EB and NBM (MDS-no EB vs MDS-EB *p*: 0.017). We observed no differences in the percentages of MEP between MDS and NBM (Figure 2c). **Abbreviations:** NBM- Normal Bone Marrow; MDS-no EB- MDS with no Blast Excess; MDS-EB- MDS with Blast Excess.

Median age in the MDS group was 74 years. Twenty-nine were MDS with no blast excess (MDS-no EB) and 9 MDS with blast excess (MDS-EB). Median age of normal controls was 67 years old. The proportion among male and female was comparable (Table 1). To analyze ROS levels among the different MDS cells subpopulations, we examined the percentages of stem and progenitor cells in MDS samples, compared to NBM. As expected, HSC percentage was higher in MDS versus NBM samples (Figure 2a). Focusing on MDS, we showed that HSC were significantly more elevated on MDS-EB compared to MDS-no EB (Figure 2b). Regarding progenitor cells, as previously reported by Woll P et al. [2], we found that CMP were expanded in MDS no-EB versus MDS-EB and NBM; on the contrary, GMPs were increased in MDS-EB samples compared to MDS no-EB and NBM. MEPs percentages did not differ among groups (Figure 2c).

**Figure 3:**
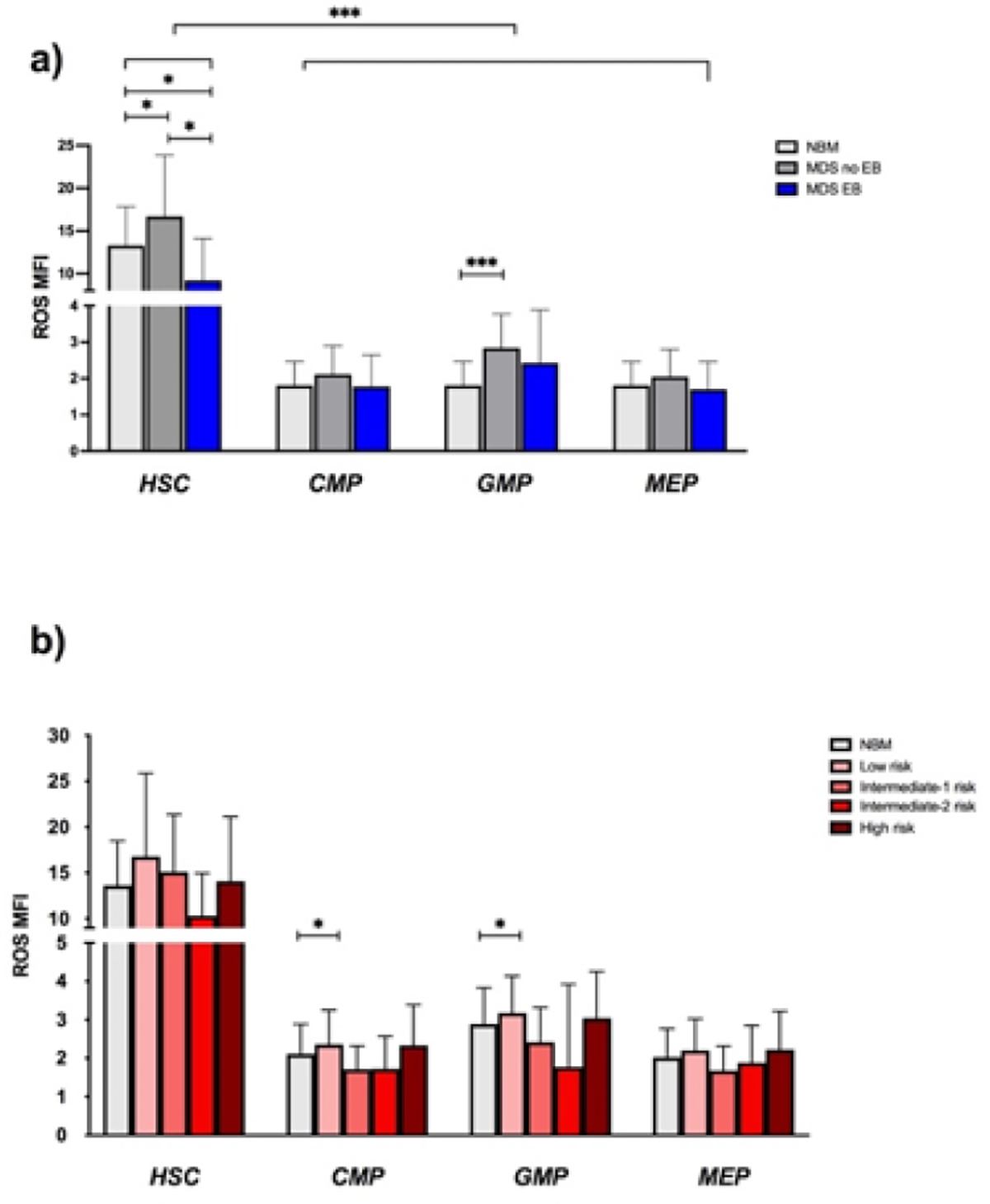
ROS levels in NBM and MDS subtypes. ROS levels in HSC were higher compared to the other subpopulations, irrespectively of the sample group (p < 0.0001). HSC from MDS-no EB express more ROS than NBM (16.6 vs 13.2, *p*: 0.03) and then MDS-EB (16.6 vs 9.2, *p*: 0.01). It is also significant the difference of ROS expression between NBM and MDS-EB HSC (13.2 vs 9.2, *p*: 0.04). Regarding progenitors, ROS levels were significantly higher in MDS-no EB GMP compared to NBM GMP, (2.8 vs 1.8, *p*: 0.01) (Figure 3a). Looking at IPSS score, low risk MDS CMP and GMP had higher ROS levels compared to NBM CMP and GMP (2.36 and 3.17 vs 1.81 and 2.58, *p*: 0.025 and *p*: 0.041) (Figure 3b) Abbreviations: MFI- Mean Fluorescence Intensity; ROS- Reactive Oxygen Species.

Next, we examined ROS levels between the different stem and progenitor cells of NBM and MDS samples. HSCs, compared to progenitors, globally showed higher ROS in all the subgroups and they were significantly more elevated in MDS-no EB, versus NBM and MDS-EB. GMP from MDS-no EB showed higher ROS compared to NBM GMP (Figure 3a). In NBM and in MDS, no correlation was seen between age and ROS expression in all the subpopulations (data not shown). Accordingly to IPSS subgroups, no differences were spotted between ROS levels on each IPSS group subpopulations. Again, HSC showed higher ROS values compared to progenitors in all IPSS risk category (Figure 3b).

**Figure 4:**
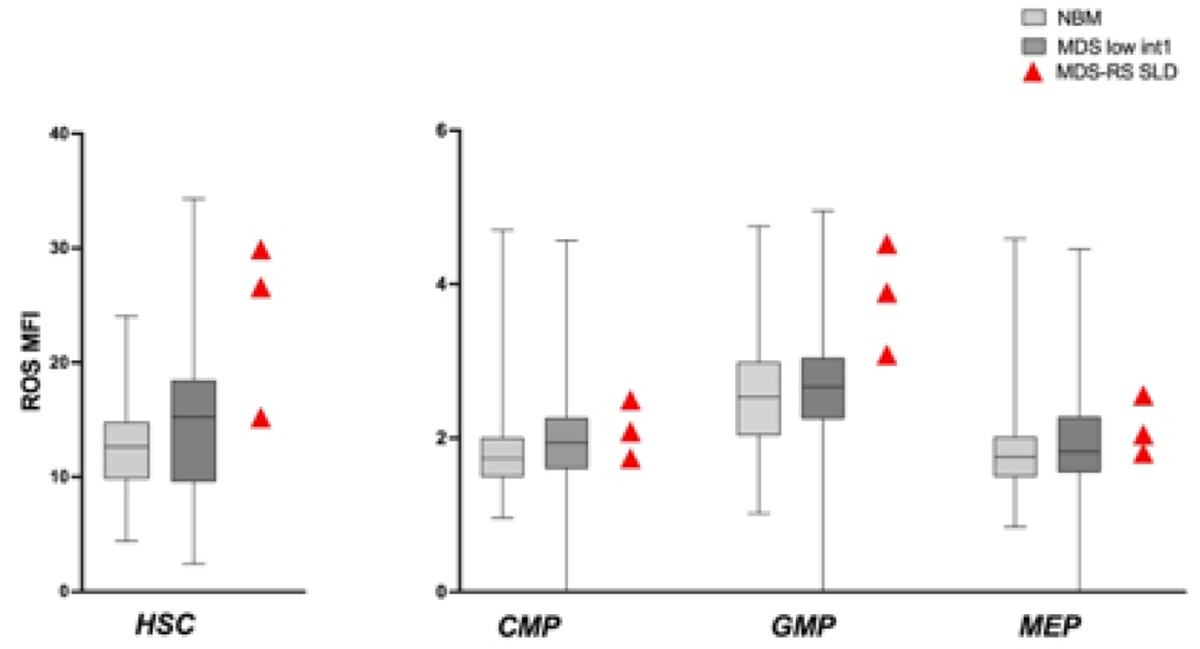
ROS in MDS-RS SLD. ROS in HSC and progenitors of 3 patients with MDS-RS-SLD. ROS levels were higher in HSC and GMP compared to NBM and low/intermediate-1 risk MDS. Box and whiskers with ROS MFI median values were used to show NBM and MDS groups.

In our MDS group, we had 3 patients with MDS with ringed sideroblasts with single lineage dysplasia (MDS-RS-SLD). In this specific subset, we noted that ROS were particularly high in HSC and in GMP, compared to those observed in NBM and in non RS low/intermediate-1 MDS (Figure 4).

**Figure 5:**
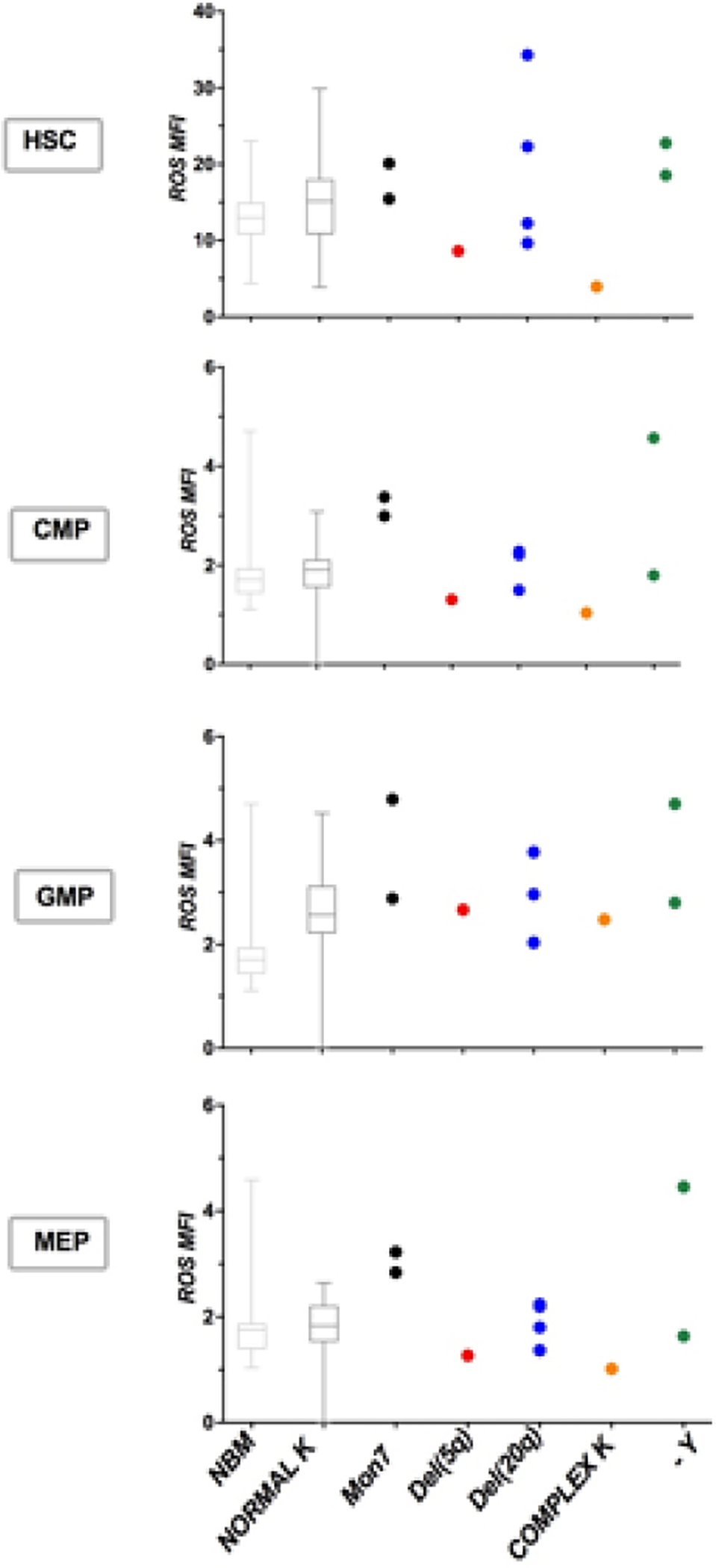
ROS and different karyotypes. We analysed ROS levels in patients with different karyotypes: 2 with monosomy 7 (Mon7), 1 with deletion of long arm of chromosome 5 (del(5q)), 3 with deletion of chromosome 20 (del(20q)), 1 with complex karyotype (complex K) and 2 with deletion of chromosome Y (-Y). NBM and MDS with normal karyotype were displayed with boxes and whiskers and ROS MFI median values.

We then looked at the different cytogenetics of MDS patients, to see whether karyotype could influence ROS expression on subpopulations. Unfortunately, we had just 2 patients with monosomy 7, 1 patient with deletion of 5q chromosome, 1 with complex karyotype, 4 patients with deletion of 20q chromosome and 2 patients with -Y. Patients with monosomy 7 showed higher ROS levels in every subpopulation, while patient with complex karyotype expressed low ROS levels in HSC and progenitor cells. The deletion 20q and -Y patients had wide ROS levels; the del5q patient displayed intermediate-low ROS values in all the subpopulations, except for GMP (Figure 5).

**Figure 6:**
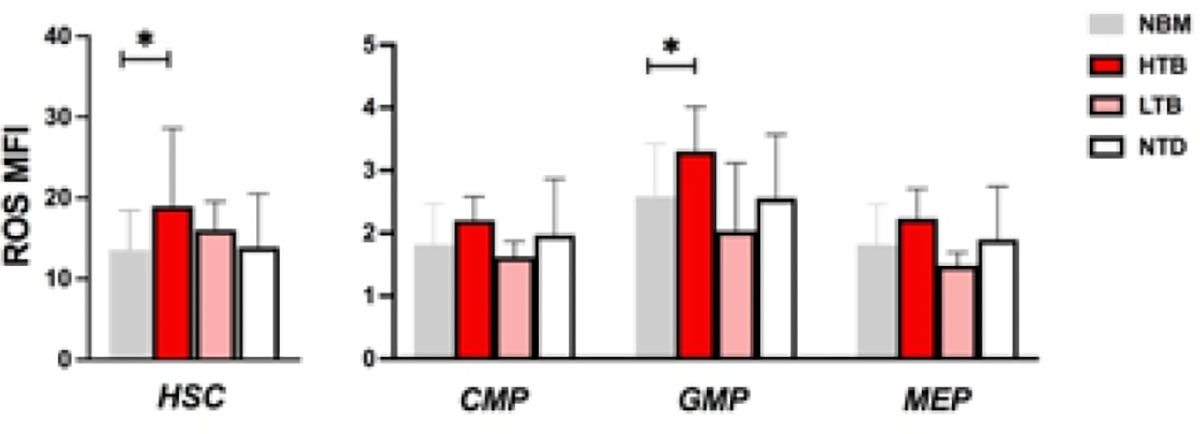
ROS levels according to patient’s transfusional status. Patients were stratified in relation to their transfusional need: high transfusion burden (HTB) (9), low transfusion burden (LTB) (2) and non-transfused patients (NTD) (26). Higher ROS levels were shown in HTB HSC and GMP compared to NBM HSC and GMP, (respectively 18.9 vs 13.6, *p*: 0.02 and 3.3 vs 2.5, *p*: 0.02).

To understand if ROS levels could influence the clinical history and evolution of MDS, we studied the correlation between ROS expression and patients’ clinical data. Looking at transfusion dependency, we analyzed all the MDS patients together, according to their transfusional status. Patients were divided in 3 groups: high transfusion burden (HTB) (n: 9), low transfusion burden (LTB, n: 2) and non-transfused patients (NTD, n:26), according to the criteria previously described [29]. We saw that patients with HTB, showed significantly higher ROS in HSC and GMP compared to NBM. Regarding NTD group, ROS levels were almost identical to ROS in NBM group (Figure 6).

**Figure 7:**
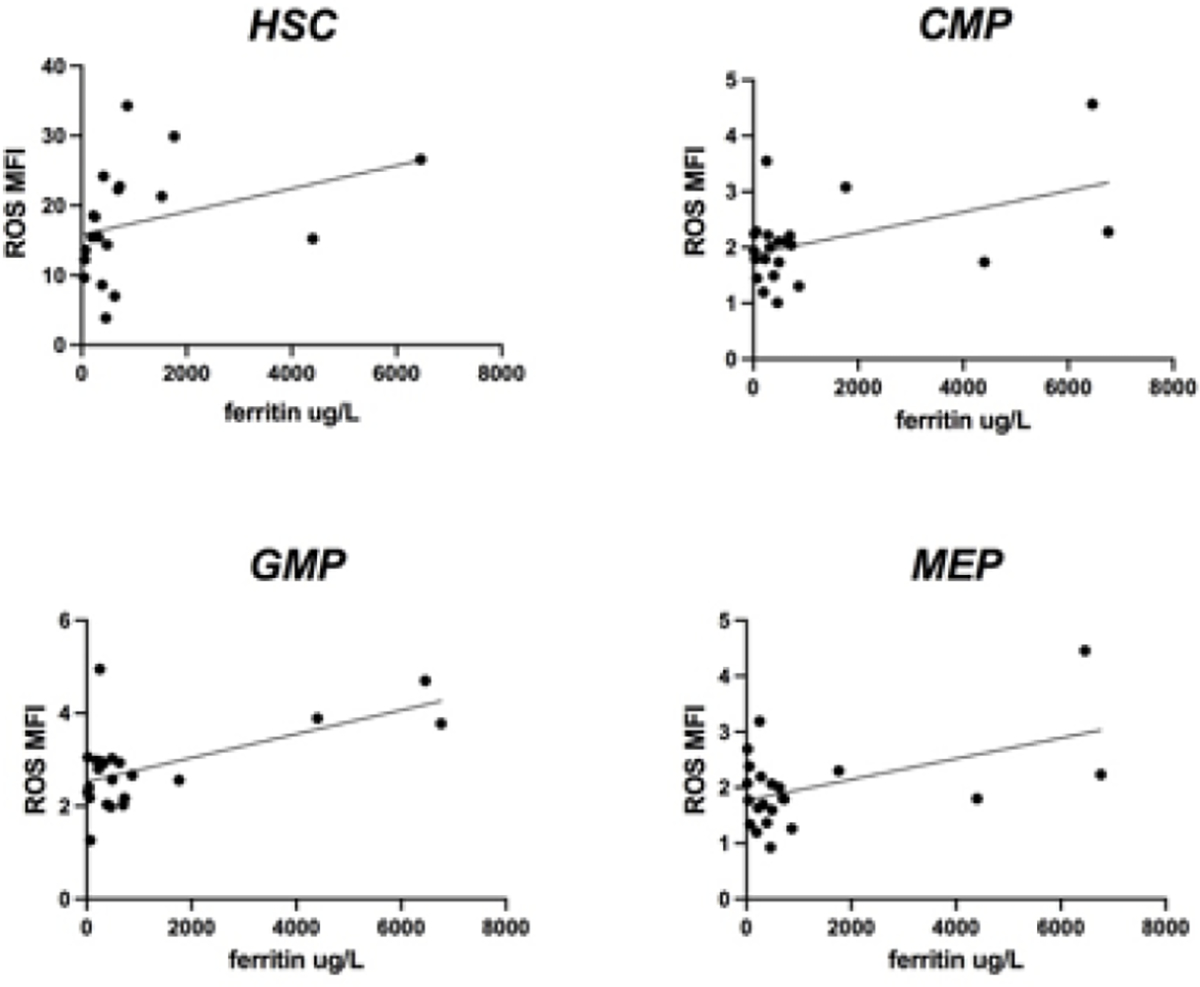
ROS levels according to ferritin values in low/intermediate 1 risk patients. A direct positive correlation was seen between CMP, GMP and MEP and serum ferritin in low/intermediate 1 patients (p: 0.024; p: 0.004; p: 0.02). No statistically significant direct correlation was shown between HSC and ferritin values.

We then evaluated ferritin values: in low/intermediate-1 MDS, as expected, a direct correlation between ferritin values and progenitors ROS levels was detected; in IPSS intermediate-2/high patients, these results were not seen. A tendency to direct correlation between ferritin values and ROS in HSC was detected but without statistical significance (Figure 7).

**Figure 8:**
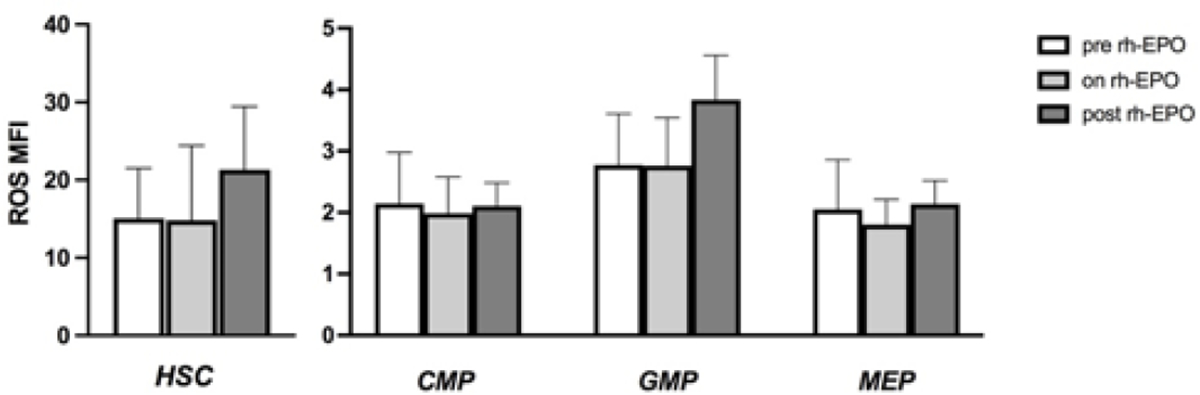
ROS levels according to rh-EPO treatment. An increased in ROS MFI was displayed in MDS patients that had stopped rh-EPO therapy especially on HSC and GMP. These results were not statistically significant. Abbreviations: rh-EPO- Recombinant Human Erythropoietin.

We, then, wanted to see if there was a correlation between ROS levels and peripheral absolute neutrophil count (ANC) in MDS patients: patients showing ANC < 1.800/ul showed lower ROS in progenitors than patients with ANC > 1.800/ul. Of note, samples cellularity did not influence ROS levels (data not shown). Subsequently, we evaluated whether therapy with recombinant human erythropoietin (rh-EPO) could influence ROS status. We sorted patients according to rh-EPO treatment. ROS levels were analyzed in patient’s pre, on and post rh-EPO. Higher ROS levels were shown on HSC of post rh-EPO low/intermediate-1 patients (Figure 8a) compared to the pre and on rh-EPO patients. Looking at the progenitor cells, GMP showed higher ROS levels in the same subset of patients (Fig. 8b), compared to CMP and MEP.

## 5. Discussion

In the last decade, what we know about MDS has largely improved. Unfortunately, our increasing biology knowledge on MDS does not always coincide to an improvement in clinical outcome. In fact, the most used treatments in MDS are still the same from the 2000s: rh-EPO is mainly used in low and intermediate-1 MDS while demethylating agents are the standard of care in intermediate-2 and high risk MDS. In the last couple of years, the anti TGF-beta agent luspatercept (ACE-536), has been proved to increase hemoglobin levels in ringed sideroblast MDS, reducing transfusional burden [30] and now it is commercially available worldwide. Since the 1980s, ROS has been studied in different hematological malignancies [31]. In cancer, oxidative stress plays a role in the pathogenesis of the disease, leading to genomic instability [32]. In MDS specifically, oxidative stress can contribute to maintain the malignant clone and influence disease progression [10]. Also, MDS cells metabolism can be involved in ROS generation. Healthy HSC use glycolysis for energy production while MDS stem and progenitor cells generate energy via oxidative phosphorylation; as a consequence, ROS levels increases [33]. In the last years, the role of inflammation was investigated in MDS pathogenesis: many studies showed an increase in pro inflammatory cytokines as IL-1β, IL-6, TNFα, and IFN-γ in low risk MDS [34]. New evidences show a link among inflammation, metabolism and clonal evolution in MDS. For example, TET2 mutated HSC can initiate leukemic clone if triggered by an inflammation microenvironment [35]; furthermore, TET2 mutation cause an increase of IL-6 in the bone marrow [23]. So, the dysregulated metabolism together with proinflammatory mechanisms can influence the clonal hematopoiesis of indeterminate potential (CHIP) emerging clones and evolution to MDS [36, 37]. In our study, we intended to investigate ROS levels in the different MDS subpopulations; it is known that ROS production can be influenced by many external factors, as time, temperature, technical procedures, so we decided to not separate CD34+ cells from bone marrow mononuclear cells, in order to avoid extra stress to cells and artificially increase ROS production. Also for this reason, analysis was run within one hour after bone marrow aspiration. ROS flow cytometry staining was performed as first step, followed by staining for hematopoietic stem and progenitor cells. To standardize results among different samples run in different days, we chose to use MFI ratio between the studied subpopulation MFI and same patient lymphocytes MFI. We showed that ROS levels and oxidative stress can be very different among the subtypes of MDS. Thanks to flow-cytometry based techniques, we have been able to confirm the structure of hematopoietic hierarchy and to compare ROS levels in different subpopulations. We saw that, especially in low risk MDS, ROS levels are higher in GMP and HSC. This factor can lead to genomic instability and can promote the expansion of the leukemic clone. In previous studies, GMP were identified as the leukemia initiating cell [38, 39] corroborating the hypothesis that GMP have a central role in clonal evolution. In low risk MDS, ROS levels are more elevated compared to the higher risks. It is also interesting that ROS ratio was higher in patients that stopped rh-EPO therapy: we think that in these patients MDS clone is more stressed than in the pre rh-EPO patients, maybe because the disease history is longer. Probably this is also associated with the loss of response to rh-EPO therapy. In MDS-RS, again, we saw an increased ROS levels more evident in GMP and HSC. In this group of MDS, mitochondrial respiration is particularly impaired and so the oxidative stress [40]. We could speculate that in the low risk MDS, chronic diserythropoiesis leads to ROS increase in both hematopoietic and progenitor populations. This process can bring to a further genomic instability and additional molecular abnormalities: in fact, secondary AML (s-AML) have more adverse cytogenetic and molecular abnormalities compared to the de-novo AML and are usually refractory to chemotherapy [41]. In low/intermediate-1 MDS, a major factor influencing oxidative stress is IO. IO can bring to a reduction of ATP/AMP ratio in MDS bone marrow cells [42] compared to normal controls. This unusual cellular respiration can bring not just to decrease in energy production but also to a ROS production and subsequent genetic instability [43]. IO can also influence cardiovascular events in MDS patients because it triggers the onset of atherosclerosis-like changes [44]. Clinically, the “TELESTO” trial showed that iron chelation therapy positively influences event free survival in low/intermediate risk MDS patients: in particular, treatment with iron chelator deferasirox significantly delayed cardiovascular secondary events in MDS patients [45]. From a molecular point of view, deferasirox leads to a partial repair of the mitochondrial function [42]. Also in our study, we saw that ROS levels are hugely influenced by IO: ROS in HSC and GMP were higher in HTB patients compared to LTB and NTB patients and directly correlated with serum ferritin values. In this scenario, where many factors can influence ROS status in MDS, there are many drugs, already available, that can impact oxidative stress in MDS and, possibly, ameliorate clinical outcomes. As we mentioned before, one of the most important treatments in low/intermediate risk MDS is iron chelation therapy that improves survival perturbing ROS status. Commercially, many other antioxidant medication, such as ascorbic acid and N-acetylcysteine (NAC), are available. These drugs are world-wide accessible at reasonable prices, as well as much tolerated by patients. In vitro reports studied the use of these antioxidant drugs on MDS CD34+ cells: NAC plus all Trans retinoic acid (ATRA) were added to granulocyte-macrophage colony forming units (CFU-GM) colonies. NAC was particularly efficient in reducing apoptosis and increasing CFU-GM colonies [46].

Also clinical reports of arsenic trioxide (ATO) combined with decitabine (DAC) showed an increase of high-risk MDS cells apoptosis via ROS production [47]. It has been reported that patients with hematological malignancies, including MDS, are vitamin-C deficient [48]. Vitamin-C is essential for TET-induced conversion of 5-methylcytosine (5mC) to 5-hydroxymethylcytosine (5hmC), that is the first step of DNA methylation [49]. Studies showed that prescribing vitamin C to MDS patients undergoing azacytidine (Aza) treatment could increase 5hmC/5mC ratio and so may amplify Aza clinical effect [50]. In the last years, the B cell lymphoma 2 (Bcl-2) inhibitor Venetoclax (VEN) has emerged as an important therapeutic option in many hematologic malignancies. VEN was shown to influence ROS balance in high risk MDS cells [51]. Many clinical trials studied the combo VEN plus Aza or DAC in AML and high risk MDS: the results are positive, but, mostly in MDS patients, a considerable high ratio of adverse events was seen [52]. To our knowledge, there are no trials of Bcl-2 inhibitors in low/intermediate risk MDS. We know that in AML the combination VEN-Aza suppress oxidative phosphorylation targeting leukemia stem cells (LSC) [53]. So, this treatment can select LSC on a ROS level basis. Importantly, normal HSC compensate the decreased oxidative phosphorylation via glycolysis increment [51], escaping the cytotoxic effect of VEN-Aza. In conclusion, our study evaluated how ROS levels diverged in the MDS hematopoietic stem and progenitor cells. In particular, the HSC and GMP seem to be the more “stressed” cells in the MDS microenvironment; nevertheless, in most cases of acute myeloid leukemia, the primary AML CD34+ cell belongs to these two subpopulations. ROS status can significantly influence the genetic stability of MDS progenitor and HSC. In this scenario, we intend to emphasize the importance of an accurate chelation therapy, in order to reduce oxidative stress among MDS bone marrow microenvironment. Actual researches are investigating therapies that act in regulating oxidative stress and hematopoietic cells metabolism, as the anti Bcl2 drugs, the ascorbic acid and NAC. Further laboratory studies are needed to understand how ROS can increase the risk of AML transformation; moreover, clinical protocols using antioxidant drugs together with standard regimens may be helpful in giving MDS patients a clinical benefit and ameliorate their prognosis and quality of life.

## Figures and Tables

**Table 1:** patients and controls characteristics.

	n	%
**PATIENTS**	38	
AGE	75 (22–89)	
MALE/FEMALE	23/15	
		
**Diagnosis acc. to WHO 2016**		
MDS-SLD	11	29
MDS-MLD	14	37
MDS-EB-1	6	16
MDS-EB-2	3	8
MDS-RS-SLD	3	8
Del(5q)	1	3
		
**IPSS**		
LOW	17	45
INTERMEDIATE 1	15	39
INTERMEDIATE 2	2	5
HIGH	4	11
		
**KARYOTYPE**		
Normal	25	66
-Y	2	5
-7	2	5
Tris15	1	3
Del(20q)	4	11
Del(5q)	1	3
Del(9q)	1	3
Complex	1	3
NA	1	3
		
**NORMAL CONTROLS**	27	
AGE	65 (30–75)	
MALE/FEMALE	16/11	

**Abbreviations:** MDS-SLD- MDS with Single Lineage Dysplasia; MDS-MLD- MDS with Multilineage Dysplasia; MDS-EB-1- MDS with Excess Blasts 5–10%; MDS-EB-2- MDS with Excess Blasts 10–20%; MDS-RS-SLD- MDS with Ring Sideroblasts and Single Lineage Dysplasia; Del(5q)- MDS with Isolated Del(5q); -Y- Chromosome Y Deletion; -7- Monosomy of Chromosome 7; tris15- Trisomy of Chromosome 15; del(20q)- Deletion of Chromosome 20q; del(9q)- Deletion of Chromosome 9q; complex- more or Equal to 3 Independent Cytogenetic Abnormalities; NA- Not Available.
